# *De novo* deletions and duplications of 17q25.3 cause susceptibility to cardiovascular malformations

**DOI:** 10.1186/s13023-015-0291-0

**Published:** 2015-06-14

**Authors:** F. J. Probst, R. A. James, L. C. Burrage, J. A. Rosenfeld, T. P. Bohan, C. H. Ward Melver, P. Magoulas, E. Austin, A. I. A. Franklin, M. Azamian, F. Xia, A. Patel, W. Bi, C. Bacino, J.W. Belmont, S. M. Ware, C. Shaw, S.W. Cheung, S. R. Lalani

**Affiliations:** Department of Molecular and Human Genetics, Baylor College of Medicine, One Baylor Plaza, MS BCM225, Houston, TX USA; Department of Neurology, Memorial Hermann Texas Medical Center, Houston, TX USA; Genetic Center, Children’s Hospital Medical Center Of Akron, Akron, OH USA; Department of Developmental Pediatrics, Texas Children’s Hospital, Houston, TX USA; Departments of Pediatrics and Medical and Molecular Genetics, Indiana University School of Medicine, Indianapolis, IN USA

**Keywords:** 17q25 Deletion, Copy number variations, Congenital heart defects, Intellectual disability

## Abstract

**Background:**

Genomic disorders resulting from deletion or duplication of genomic segments are known to be an important cause of cardiovascular malformations (CVMs). In our previous study, we identified a unique individual with a *de novo* 17q25.3 deletion from a study of 714 individuals with CVM.

**Methods:**

To understand the contribution of this locus to cardiac malformations, we reviewed the data on 60,000 samples submitted for array comparative genomic hybridization (CGH) studies to Medical Genetics Laboratories at Baylor College of Medicine, and ascertained seven individuals with segmental aneusomy of 17q25. We validated our findings by studying another individual with a *de novo* submicroscopic deletion of this region from Cytogenetics Laboratory at Cincinnati Children’s Hospital. Using bioinformatic analyses including protein-protein interaction network, human tissue expression patterns, haploinsufficiency scores, and other annotation systems, including a training set of 251 genes known to be linked to human cardiac disease, we constructed a pathogenicity score for cardiac phenotype for each of the 57 genes within the terminal 2.0 Mb of 17q25.3.

**Results:**

We found relatively high penetrance of cardiovascular defects (~60 %) with five deletions and three duplications, observed in eight unrelated individuals. Distinct cardiac phenotypes were present in four of these subjects with non-recurrent *de novo* deletions (range 0.08 Mb–1.4 Mb) in the subtelomeric region of 17q25.3. These included coarctation of the aorta (CoA), total anomalous pulmonary venous return (TAPVR), ventricular septal defect (VSD) and atrial septal defect (ASD). Amongst the three individuals with variable size duplications of this region, one had patent ductus arteriosus (PDA) at 8 months of age.

**Conclusion:**

The distinct cardiac lesions observed in the affected patients and the bioinformatics analyses suggest that multiple genes may be plausible drivers of the cardiac phenotype within this gene-rich critical interval of 17q25.3.

**Electronic supplementary material:**

The online version of this article (doi:10.1186/s13023-015-0291-0) contains supplementary material, which is available to authorized users.

## Background

Several rare recurrent DNA copy number variations (CNVs) and novel genomic loci have been implicated in congenital cardiac malformations, categorically establishing the importance of CNVs in clinical evaluation of individuals with CVM [1–5]. It is estimated that genomic disorders account for approximately 10 % of all CVM cases. While 22q11.2 deletion (MIM 188400) remains the most common genomic disorder responsible for CVM, other less frequent, but important, contributors include 1p36 monosomy (MIM 607872), Williams-Beuren syndrome (7q11.2 deletion; MIM 194050), 8p23.1 deletion encompassing *GATA4* and *SOX7,* 9q34 deletion involving *EHMT1* (MIM 610253), and 17q21.31 microdeletion including *KANSL1* (MIM 610443). These genomic disorders are known to be associated with relatively high penetrance of CVM, often affecting dosage sensitive genes within the deleted intervals. The recurrent 1q21.1 distal deletion (Class I deletion; MIM 612474) encompassing *GJA5* is also associated with CVM [6] with incomplete penetrance (10–25 % of cases). The reciprocal duplication 1q21.1 (MIM 612475), on the other hand is strongly linked to tetralogy of Fallot (TOF) in several studies [2, 7, 8]. The recurrent 22q11.2 distal deletion is yet another genomic disorder linked to cardiac malformations in numerous reports [9, 10]. In our previous study of over 700 individuals with syndromic CVMs, we identified a *de novo* submicroscopic 17q25.3 loss in an affected individual, which was not observed in over 2,800 controls [1]. Other than this unique case, pure subtelomeric deletions confined to 17q25.3 have not been reported. Pure 17q25.3 submicroscopic copy number gains are also infrequent, and have been observed in association with distal arthrogryposis, craniofacial dysmorphism and atrial septal defect (ASD) [11]; intellectual disability [12, 13]; and with severe microcephaly with concurrent mutation in *WDR62* [14]. While several reports of unbalanced rearrangements of terminal 17q have been described with concomitant monosomy or trisomy of other autosomes and X chromosome [15–21], pure subtelomeric rearrangements of 17q25.3 unarguably remain inadequately delineated amongst the group of subtelomeric disorders. Here we report a case series of eight individuals, five with pure non-recurrent submicroscopic 17q25.3 deletions and three with 17q25 duplication. We provide a detailed phenotypic characterization associated with genomic rearrangement of this region, and show a penetrance of ~60 % for CVMs in this group. This study identifies a novel genomic locus responsible for congenital cardiac malformations and identifies potential critical genes within the terminal region of 17q25.3 related to cardiac morphogenesis.

## Subjects and methods

### Human subjects

Patients were ascertained from screening of 60,000 samples submitted for clinical chromosomal microarray analyses completed at the Medical Genetics Laboratories (MGL) of Baylor College of Medicine (BCM). The study was performed in accordance with the institutional guidelines for human research with approval by the Institutional Review Board of Baylor College of Medicine and Cincinnati Children’s Hospital Medical Center. The novel genomic loss of subject 1 was previously reported in the literature [1]. Photographs were obtained for publication after appropriate parental consents.

### Cytogenetic, molecular cytogenetic and molecular analysis

DNA was extracted from whole blood by the Puregene DNA Blood Kit (Gentra) according to the manufacturer’s instructions. The procedures for DNA digestion, labeling, and hybridization for the oligo arrays were performed according to the manufacturers’ instructions. Seven of the eight subjects were studied with custom-designed genome-wide array with approximately 180,000 oligonucleotides, manufactured by Agilent Technologies, Inc. (Santa Clara, CA) as previously described [22]. The clinical array is designed by the MGL at BCM with exon-by-exon coverage for about 1,700 genes and 700 microRNAs. Confirmatory FISH analyses for 17q25 deletion were performed using RP11-497H17 and RP11-1182P23. Subject 3 was studied in the Cytogenetics Laboratory at Cincinnati Children’s Hospital using both bacterial artificial chromosome (BAC) and single nucleotide polymorphism (SNP) arrays. The SignatureSelect V2 chip containing approximately 4671 BAC clones concentrated in areas of clinical significance, was used for array-CGH. Additional analysis was performed using the Infinium Assay with the Illumina HD Human610-quad BeadChip platform containing approximately 620,900 markers.

### Gene ontology, gene expression, protein-protein interaction studies

To identify the cardiac-specific genes within the terminal region of 17q25, we utilized GeneOntology, Gene Expression, Protein-Protein Interaction networks, haploinsufficiency scores, and miRNA targeting information to score 57 genes encoded within the terminal 2 Mb region of 17q25.3 for relevance to the cardiac phenotype. This approach has successfully been used in our previous studies in identifying novel genes underlying CVMs and epilepsy [1, 23].

We analyzed the possibility of CNV loss of each gene contributing to the cardiac phenotype by developing a pathogenicity score, trained using a set of 251 cardiac specific genes that we contextualized via a composite set of annotation resources (Additional file 1: Table S1). To ensure that the constituents of the training set spanned the breadth of genes in which mutations have been observed to cause CVM, we filtered procedures on the Clinical Synopsis data available in Online Mendelian Inheritance in Man (MIM) and manually curated all disorders with any phenotype under the class Cardiovascular. We further expanded upon the training set by identifying additional genes both co-enriched (Fisher’s test) with the initial set for the same subset of descriptive terms in the Human Phenotype Ontology [24], and highly similar (Resnik method of calculating semantic similarity) to the initial set [25]. The annotation content employed comprised protein-protein interaction (PPI) data, human tissue expression patterns, microRNA (miRNA) targeting, haploinsufficiency scores [26], known gene-to-disease relationships in the MIM database [27], and phenotype annotations in the Gene Ontology (GO) [28] and Mammalian Phenotype Ontology (MPO) [29].

Our scores were determined from the ranked sum of feature scores for each candidate gene. To help ensure that contributions of features were proportionate to their variability and measurement scale, we calculated weighting coefficients from the coefficient of variation of measurements within each feature, most heavily weighing features with the largest amount variability across genes relative to their mean. We also computed the rank of sums of the unweighted candidate feature scores. Two of these features were binary, indicating Yes/No as to whether a candidate gene has been observed in a reported variant as causing heart phenotype in mice (MPO) or in MIM. Another of these features, haploinsufficiency, is itself a previously developed phenotypically aggregate score of developing deleterious phenotypes in the presence of only a single copy of a candidate gene [26]. We also calculated as a feature, a T-statistic scoring candidate gene expression differences between the 10 tissues expressing training genes at the highest levels and the 10 tissues expressing training genes at the lowest levels. In addition, we included as a feature the ontological enrichment of each gene measured against the categories significantly enriched in annotations to the training genes compared to the background using a measure of overlap of GO annotation categories that was determined for each candidate gene. Finally, we calculated as another feature the protein-interaction network communicability of each candidate gene to the training genes normalized to the typical communicability of each gene to the background using the InWeb Protein Interaction Database [30] (Additional file 2: Table S2).

## Results

### Subject 1

**Fig. 1:**
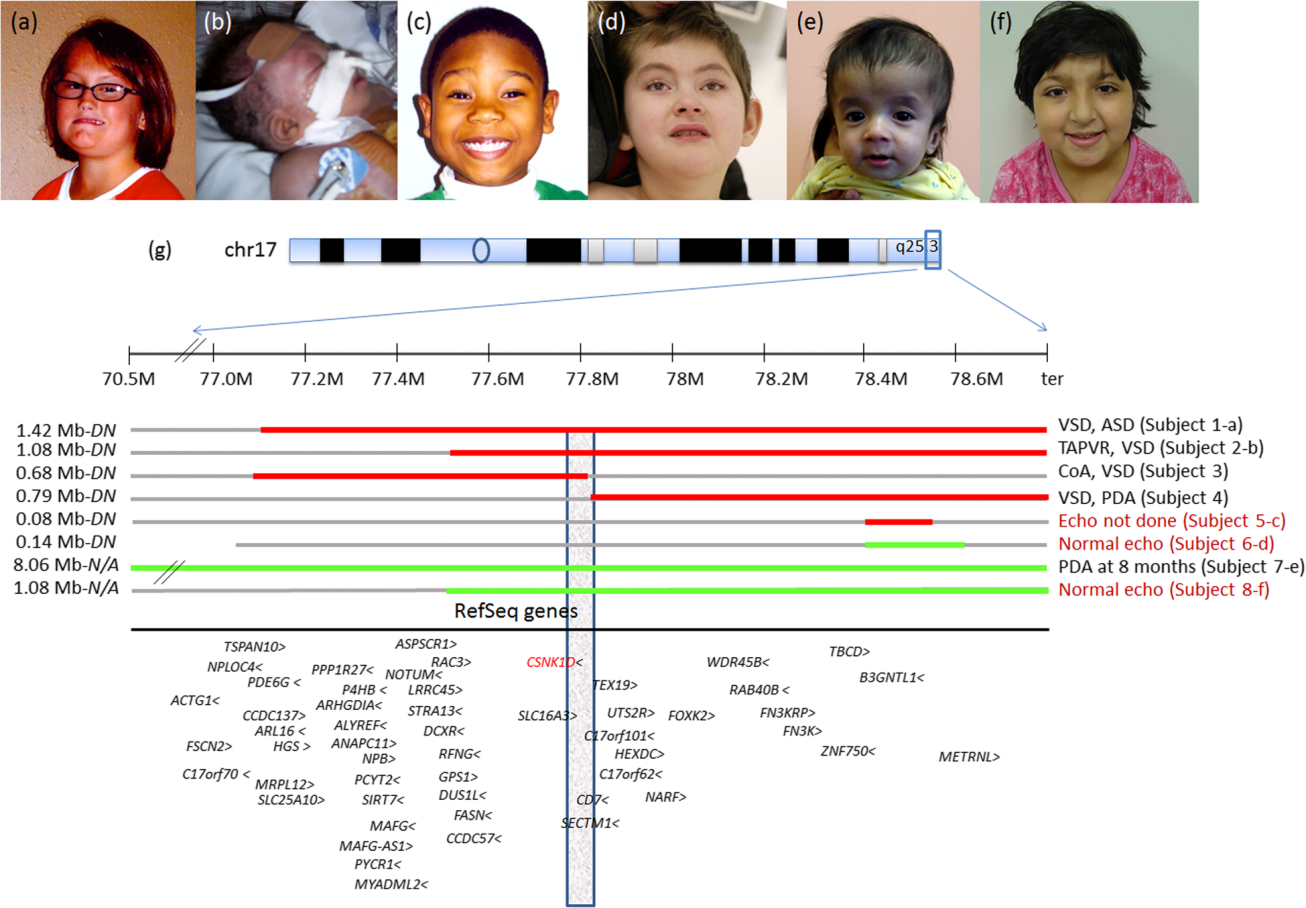
Breakpoint mapping in eight subjects with 17q25 deletions and duplications (based on hg18). The red bars indicate deletion and green bars represent duplications. The first three photos (**a**, **b**, **c**) represent subjects with deletion. Individuals with duplication are shown in panels, **d**, **e** and **f**. Note the variability of phenotype related to genomic rearrangements in the group. Notably, when parents were tested, all events were apparently *de novo* (DN) in origin

Subject 1 was diagnosed with perimembranous ventricular septal defect (VSD) and atrial septal defect (ASD) around one week of age, after she presented with congestive heart failure. She was treated medically with spironolactone, digoxin, and furosemide and was transitioned off her cardiac medications by the age of 2 years. Repeat echocardiogram at 4 years of age revealed closure of the septal defects. Her additional medical problems included strabismus, early feeding difficulties, gastroesophageal reflux, and recurrent otitis media. She was diagnosed with mixed receptive-expressive language disorder, articulation disorder and borderline intellectual functioning at 8 years of age. Her physical examination was remarkable for normal growth parameters of weight of 27.9 kg (50th-75th percentile), height of 122.4 cm (10th-25th percentile), and head circumference of 54.0 cm (50th-90th percentile), facial dysmorphism including upslanting palpebral fissures, midface hypoplasia, downturned corners of the mouth (Fig. 1a), mild scoliosis, and short tapered digits.

The G-banded karyotype analysis and fragile X studies were normal. The array CGH revealed a *de novo* copy number loss in the subtelomeric region of 17q25.3 of approximately 1.425 Mb in size, confirmed by FISH analysis. The proximal breakpoint mapped between 77,173,756 and 77,213,237 (hg18) for this terminal deletion including over 40 RefSeq genes.

### Subject 2

Subject 2 was diagnosed with infra-diaphragmatic total anomalous pulmonary venous return (TAPVR) immediately after birth. He was born at 36 weeks gestation to a 26-year-old female with a history of two prior spontaneous abortions at 6 weeks. The pregnancy was complicated by intrauterine growth restriction. The birth weight was 1734 g (<3rd percentile), birth length was 43 cm (10^th^ percentile), and head circumference was 30 cm (5–10^th^ percentile). Other notable features included left eyelid coloboma, tall sloping forehead, smaller right ear in comparison to the left, high-arched palate, triangular shaped face, single transverse palmar crease on the left, rocker bottom feet, and sacral dimple (Fig. 1b). He had a complicated post-operative course requiring extracorporeal membrane oxygenation (ECMO), and passed away on day of life 13. Autopsy revealed left atrial and ventricular hypoplasia, ASD (fenestrated secundum type), VSD, bicuspid aortic valve, patent ductus arteriosus (PDA), immature brain, two accessory spleens, and unilobar left lung.

**Fig. 2:**
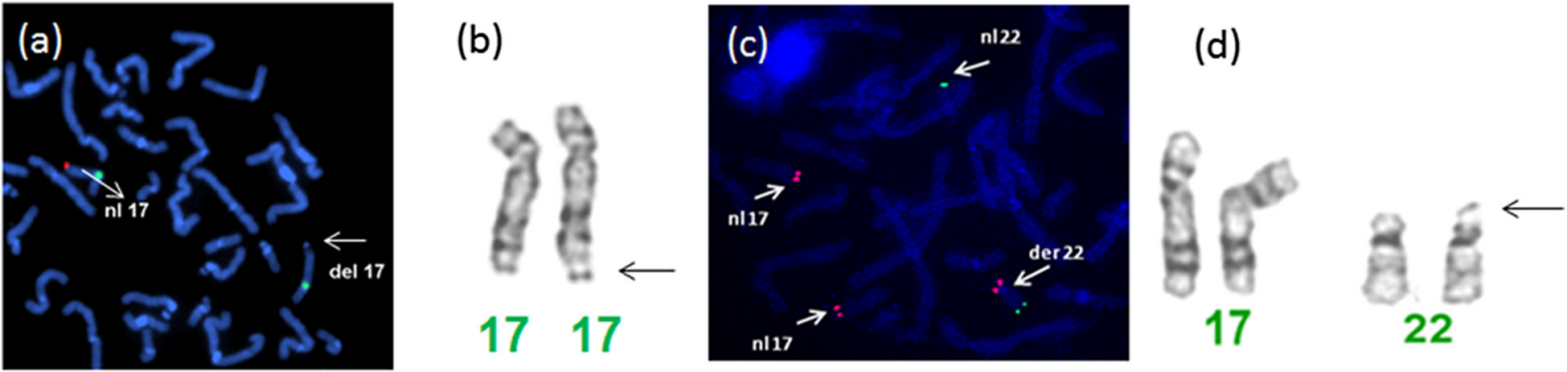
FISH and partial karyotype images in subject 2 (**a**, **b**) and subject 7 (**c**, **d**) are shown. Note the additional satellited material of unknown origin attached to the long arm of one chromosome 17 at band 17q25.3 in subject 2, resulting in *de novo* copy number loss of the terminal subtelomeric region of 17q25.3 of approximately 1.083 Mb in size. In subject 7, FISH analysis revealed a derivative chromosome 22 with the extra copy of 17qter translocated to the distal short arm of chromosome 22 (**c**), also observed retrospectively on partial karyotype study

Karyotype study showed 46,XY,add(17)(q25.3), with additional satellited material of unknown origin attached to the long arm of one chromosome 17 at band 17q25.3 (Fig. 2a, b). Array CGH revealed a *de novo* copy number loss of terminal subtelomeric region of 17q25.3 of approximately 1.083 Mb in size, with proximal breakpoint between 77,546,315 and 77,555,228.

### Subject 3

Subject 3 was diagnosed with coarctation of the aorta (CoA), multiple muscular VSDs, a perimembranous VSD, and unilateral cleft lip in the newborn period. Brain MRI was significant for the presence of ectopic neurohypophysis adjacent to the hypothalamus. He had additional diagnoses of submucus cleft palate, conductive hearing loss, subglottic stenosis, laryngomalacia, gastroesophageal reflux disease with possible intermittent aspiration, and recurrent croup and upper respiratory tract infections. No significant dysmorphic features were noted on evaluation. At 22 months, he underwent bilateral orchiopexy for undescended testicles and complex circumcision revision. He was noted to have mild glanular hypospadias with urethral meatal stenosis. At 29 months, he underwent right nasolacrimal duct stenting. He had fine and gross motor delays and received occupational and physical therapies. He was enrolled in a school for the deaf due to severe speech apraxia. At 4 years, 8 months of age, his growth parameters showed a height at the 22^nd^ percentile and weight at the 12^th^ percentile.

Array CGH showed a *de novo* copy number loss of approximately 684 kb in size within the 17q25.3 region (77,125,528-77,809,659), sparing the distal segment.

### Subject 4

Subject 4 was born at 32 weeks gestation with birth weight of 1130 g (<3^rd^ percentile) and length of 38.7 cm (10^th^ percentile). The neonatal course was complicated by tracheoesophageal fistula and tethered cord. Echocardiogram showed VSD and PDA. Brain imaging was consistent with agenesis of the corpus callosum. Her development was globally delayed when evaluated at 12 years. She had normal growth parameters with weight of 37.5 kg (34th percentile), length of 148.5 cm (44th percentile), and head circumference of 54.6 cm (50th percentile), sparse eyebrows laterally, bulbous nose, notching of alae nasi bilaterally, long digits, and mild contractures of distal lower extremities.

Array CGH showed a *de novo* loss of terminal 17q25.3 of approximately 0.80 Mb, with the proximal breakpoint mapping between 77,799,839 and 77,842,711.

### Subject 5

Subject 5 was referred for a developmental evaluation at 30 months of age due to language delay. His cardiac exam was normal, and echocardiogram was not performed. Brain MRI was significant for Chiari I malformation. Formal developmental testing using the Griffith Mental Developmental Scale revealed global developmental delay. There were no significant facial dysmorphisms noted on physical examination (Fig. 1c).

The karyotype analysis and fragile X study were normal. He was found to have a *de novo* subtelomeric loss on 17q25.3, confirmed by FISH analysis, spanning approximately 0.082 Mb, disrupting only two genes, *TBCD (Beta tubulin cofactor-D)* and *B3GNTL1*, leaving the distal *METRNL* gene intact. The proximal breakpoint mapped between 78,452,326 and 78,454,234, and the distal breakpoint mapped between 78,536,478 and 78,553,241.

### Subject 6

Subject 6 was born at term via cesarean section for fetal distress. Bilateral congenital cataracts and hypogonadism were noted at birth. His echocardiogram was found to be normal. He was additionally noted to have polysplenia. Brain MRI was significant for thinning of the corpus callosum and cortical dysplasia. At 3 years of age, he was non-verbal and non-ambulatory with global developmental delay. On physical examination, facial dysmorphic features were noted including long narrow face, small upslanting palpebral fissures, a narrow nasal bridge, and slightly prominent ears (Fig. 1d). Contractures were observed throughout, with limited extension of the lower extremities.

Karyotype study was normal. The array CGH revealed a *de novo* gain in copy number in the subtelomeric region of 17q25.3 spanning a minimum of 141 kb involving the *TBCD* and *B3GNTL1* genes, sparing the distal *METRNL* gene. The proximal breakpoints mapped between 78,457,408 and 78,458,509 and the distal breakpoint mapped between 78,599,991 and 78,623,171.

### Subject 7

Subject 7 was found to have PDA at eight months of age. His additional problems included failure to thrive and dysmorphic facial features. Brain MRI showed mild to moderate cerebral volume loss and minimal cerebellar volume loss. Physical examination at the age of 18 months showed relative macrocephaly, a large anterior fontanel, triangular facies, a prominent forehead, hypertelorism, down-slanting palpebral fissures, and low-set ears (Fig. 1e).

The karyotype was 46,XY at 500-band resolution. The array CGH revealed a gain in copy number in the 17q25.1-q25.3 terminal region, spanning approximately 8.068 Mb. The proximal breakpoint mapped between 70,528,836 and 70,570,936. FISH analysis revealed a derivative chromosome 22 with the third copy of the 17q25.1-q25.3 region translocated to the distal short arm of chromosome 22 (Fig. 2c, d). There was no evidence of a rearrangement in the mother by FISH analysis. Father’s sample was unavailable for testing.

### Subject 8

Subject 8 was evaluated at 8 years of age for intellectual disability and microcephaly. She was born with multiple cranial suture synostosis and underwent fronto-orbital advancement in early childhood. Her medical history was also significant for adjustment disorder with mixed anxiety and attention deficit hyperactivity disorder (ADHD). Her echocardiogram was normal. Brain MRI showed minimal patchy frontal encephalomalacia bilaterally. Her ophthalmological exam was unremarkable. Physical examination at 8 years was consistent with microcephaly with head circumference of 48.8 cm (<5^th^ percentile), normal weight, 31.6 kg (80^th^ percentile), and height 126.1 cm (25^th^ percentile). She was noted to have malar hyperplasia, high arched palate, micrognathia, prominent nasal bridge and columella (Fig. 1f). Neurological exam was consistent with mild hypotonia.

The array CGH revealed a gain in copy number in the 17q25.3 terminal region, spanning approximately 1.08 Mb. The proximal breakpoints mapped between 77,546,315 and 77,555,228. Parents were unavailable for further testing.

### Annotation analysis of the region

Phenotype specific pathogenicity evaluation for annotation features determined relative scores for each gene in the region, with respect to cardiac phenotype. These scores provide an algorithmic basis for prioritizing genes for subsequent functional inquiry. Each feature’s variability across genes in the region is a necessary condition for distinguishing among the genes, and the features with the largest coefficients of variation (defined as sd/mean) across all genes in the region were: known association with heart phenotype(s) in MPO, known causal association(s) between hosted variants and MIM disease and direct interactions [31]. After combining the scores to produce an aggregate result for each gene, we found that *ACTG1* and *ARHGDIA* were the highest scoring genes while *MIR3186*, *OXLD1*, *MIR6786*, *MAFG-AS1*, *MIR6787*, *OGFOD3*, and *WDR45B* all shared the equally lowest score. Detailed information is provided in Additional file 2: Table S2.

## Discussion

Our study describes eight individuals with deletions and duplications of 17q25, accentuating the occurrence of congenital cardiac abnormalities in ~60 % of subjects (5/8). The craniofacial characteristics and additional congenital anomalies of the described individuals are not typically distinguishing, possibly due to the unique structural variations, occurring in a highly gene-rich region of 17q25. Neurocognitive deficits were noted in all individuals beyond one year of age, with language delay frequently observed. Brain imaging abnormalities such as cerebral volume loss, white matter changes and corpus callosum abnormalities were noted in 6/8 individuals. Other highly variable non-cardiac anomalies included cleft palate, eyelid coloboma, cataracts, tethered cord, and musculoskeletal abnormalities (Table 1).

**Table 1:** Clinical features of eight subjects with non-recurrent deletions and duplications of 17q25

Subject	1	2	3	4	5	6	7	8
Gender	Female	Male	Male	Female	Male	Male	Male	Female
Event	Deletion	Deletion	Deletion	Deletion	Deletion	Duplication	Duplication	Duplication
Origin	*de novo*	*de novo*	*de novo*	*de novo*	*de novo*	*de novo*	N/A	N/A
Minimum Size	1.42 Mb	1.08 Mb	0.68 Mb	0.796 Mb	0.08 Mb	0.14 Mb	8.06 Mb	1.08 Mb
Echo findings	Perimembranous VSD and ASD	TAPVR, VSD, ASD, PDA, left atrial and ventricular hypoplasia, BAV	CoA, multiple muscular VSDs, perimembranous VSD	VSD, PDA	Not done	Normal study	PDA at 8 months of age	Normal study
Cytoband	17q25.3	17q25.3	17q25.3	17q25.3	17q25.3	17q25.3	17q25.1-q25.3	17q25.3
Start position (hg18)	77,173,756-77,213,237	77,546,315-77,555,228	77,125,528	77,799,839-77,842,711	78,452,326- 78,454,234	78,457,408- 78,458,509	70,528,836- 70,570,936	77,546,315-77,555,228
End position (hg18)	78,638,511-78,774,742	78,638,511-78,774,742	77,809,659	78,638,511-78,774,742	78,536,478-78,553,241	78,599,991- 78,623,171	78,638,511-78,774,742	78,638,511-78,774,742
Age at Last Examination	8 years	2 weeks	5 years, 1 month	12 years	2 years, 6 months	7 years, 5 months	1 year, 7 months	8 years
Brain Imaging	Not done	Diffuse and severe cerebral edema	Ectopic neurohypophysis, adjacent to the hypothalamus	Agenesis of corpus callosum	Chiari I malformation	Thinning of the corpus callosum and cortical dysplasia	Mild to moderate global volume loss	Minimal patchy frontal encephalomalacia bilaterally, linear focus of increased FLAIR signal in left periatrial white matter
Eye findings	Strabismus	Left eyelid coloboma	Right nasolacrimal duct obstruction	Unknown	Normal	Bilateral congenital cataracts	Mild hyperopia	Normal
Muscular/ skeletal	Normal stature, mild scoliosis	Rocker-bottom feet bilaterally	Normal stature	Normal stature, bilateral calcaneon-avicular coalition	Normal stature	Limb contractures	Short stature	Normal stature
Other problems		Polysplenia, unilobar left lung	Unilateral cleft lip, submucous cleft palate, speech apraxia, moderate conductive hearing loss left ear, subglottic stenosis, laryngomalacia, GERD, bilateral undescended testes, glanular hypospadias	TE fistula, tethered cord		Polysplenia, nocturnal hypoventilation		ADHD, psychiatric disorder

Despite a wide phenotypic spectrum observed in this group, the moderate penetrance of CVM is very compelling. The penetrance of cardiac defects is particularly higher in individuals with deletions (4/5) as compared to those with duplications (1/3). Of the five individuals with non-recurrent deletions (ranging from 0.08 Mb–1.42 Mb), all were *de novo* with four having distinct cardiac lesions including TAPVR, CoA, and septal defects. The smallest deletion in association with CVM was seen in subject 3, with the ~0.68 Mb loss encompassing at least 27 RefSeq genes. While four out of five individuals in our cohort with 17q25.3 deletions had CVMs, they did not share a commonly deleted minimal region (Fig. 1). This may suggest that multiple genes within the terminal ~2 Mb of 17q25.3 are drivers of cardiac patterning in humans. Findings suggestive of abnormal laterality were observed in two individuals; subject 2 with polyspenia and unilobar left lung; and subject 6 with polysplenia and normal echocardiogram.

None of the genes within this interval is currently implicated in human CVM, but cardiac expression is observed for several of these genes, including *ACTG1, P4HB, ARHGDIA, NPLOC4, MRPL12, DCXR, CSNK1D*, *SLC16A3* and *STRA13* (Additional file 2: Table S2).

To refine cardiac-specific genes within this locus, we used a bioinformatics approach using Gene Expression, GeneOntology, Protein-Protein Interaction networks, haploinsufficiency data, and MPO, and utilized a set of over 250 cardiac-specific genes to assign pathogenicity score to the 57 genes within the terminal 2 Mb of 17q25 region. Based on the complex computation analyses, high priority candidate genes shared by at least 3 individuals with CVMs included *ARHGDIA, MAFG, CSNK1D, RAC3, HGS,* and *SIRT7* (Additional file 2: Table S2). Other genes such as *NPLOC4, SLC16A3,* and *UTS2R* are also important considerations. It is notable that within this region, none of the deletions include *ACTG1*, which is implicated in Baraitser-Winter syndrome 2, an autosomal dominant disorder characterized by neuronal migration defect, distinctive face, and cardiac defects including bicuspid valve, VSD and PDA [32, 33] (Table 2).

**Table 2:** MIM annotated genes with known phenotype within the terminal 2.0 Mb segment of 17q25.3

Gene	Annotated MIM entries	MIM IDs	Inheritance	Heterozygous deletion and duplication in subjects
*ACTG1*	Baraitser-Winter syndrome 2; Deafness, autosomal dominant 20/26	604717; 614583	AD	**7**
*FSCN2*	Retinitis pigmentosa 30	607921	AD	**7**
*PDE6G*	Retinitis pigmentosa 57	613582	AR	**1, 3, 7**
*ARHGDIA*	Nephrotic syndrome, type 8	615244	AR	**1, 3, 7**
*PYCR1*	Cutis laxa, autosomal recessive, type IIB; Cutis laxa, autosomal recessive, type IIIB	612940; 614438	AR	**1, 3, 7**
*ASPSCR1*	Alveolar soft-part sarcoma	606243		**1, 2, 3, 7**, 8
*DCXR*	Pentosuria	260800	AR	**1, 2, 3, 7**, 8
*CSNK1D*	Advanced sleep-phase syndrome, familial, 2	615224	AD	**1, 2, 3, 7**, 8
*ZNF750*	Seborrhea-like dermatitis with psoriasiform elements	610227	AD	**1, 2, 4, 7**, 8

Bold numbers indicate subjects with cardiac malformations

*ARHGDIA*, encoding Rho GDP dissociation inhibitor α (RhoGDIα) was found to have a high pathogenicity score in our study. Involved in cardiac specific inhibition of Rho family protein, this gene is deleted or duplicated in 3/5 individuals with CVMs. While increased expression of RhoGDIα causes defective heart looping, poor trabeculation, impaired chamber demarcation, absence of endocardial cushion and hypocellularity [34], targeted inactivation of *Arhgdia* has been shown to cause severe proteinuria and nephrotic syndrome in mice [35]. Homozygous mutations in *ARHGDIA* have now been shown to cause nephrotic syndrome in several families [36, 37], consistent with the animal studies. This suggests that this gene is less likely to explain CVM in individuals with deletions, but may contribute to CVM when duplicated. It is interesting to note that the two subjects with duplications distal to this gene (subjects 6 and 8) had normal echocardiogram studies. *SIRT7,* a member of the sirtuin family of genes, is another candidate gene with a relatively high pathogenicity score affected in 3/5 subjects with CVMs. Sirt7-deficient mice develop progressive heart hypertrophy with an increased number of apoptotic cells in myocardium [38]. Sirt7-deficient cardiomyocytes show a reduced resistance to oxidative stress, indicating an important role of Sirt7 in the regulation of stress responses and cell death in the heart [38]. *HGS*, encoding hepatocyte growth factor–regulated tyrosine kinase substrate, is also a good candidate gene inferred from our bioinformatics analysis and involved in 3/5 subjects. HGS is known to transduce BMP signaling for proper embryonic development [39], and its disruption causes early embryonic lethality after gastrulation [40]. Another important candidate gene from our study affected in 4/5 subjects is *UTS2R,* encoding urotensin II and known to have potent vasoconstrictor effects. *UTS2R* has been shown to have a potential link to cardiac remodeling such as hypertrophy [41]. However, its role in structural heart defects remains to be elucidated. *CSNK1D* encodes an isoform of casein kinase I, a serine/threonine-specific protein kinase with important function in ciliogenesis [42]. Homozygous mice die within days of birth [43]. This gene is deleted in 3/5 subjects with CVMs, duplicated in the fourth, and could be either partially deleted or immediately flanking the deletion observed in subject 4 with VSD and PDA. The gene scores in the top 12 % in the pathogenicity score from our bioinformatics analysis and remains an excellent candidate gene for congenital cardiac defects observed in this study.

## Conclusion

The ubiquitous use of next generation sequencing technology in individuals with CVMs may ultimately identify causative gene(s) within this important CNV. Several of the genes within the 17q25.3 interval have significant number of predicted loss of function mutations in the Exome Aggregation Consortium (ExAC) database (Additional file 3: Table S3). Our study highlights a comprehensive phenotypic spectrum associated with rarely described 17q25 telomeric deletions and duplications and underscores the region as a novel cardiac-susceptibility locus.

### Availability of supporting data

The data set supporting the results of this article is included within the article and its additional files.

## Additional files

Additional file 1: Table S1. List of 251 genes linked to cardiac phenotype. Additional file 2: Table S2. Pathogenicity score (Equally_Weighted_Rank) of the genes within the 17q25.3 region, trained using a known set of over 200 genes from Table 1. Additional file 3: Table S3. Number of deleterious variants in genes within 17q25.3 from the ExAC dataset.
